# Association Between Serum SFRP1 Levels and Coronary Artery Calcification in Stage 5D Chronic Kidney Disease Undergoing Maintenance Hemodialysis

**DOI:** 10.33549/physiolres.935693

**Published:** 2026-04-01

**Authors:** Huihui CHEN, Jingjing JIN, Meijuan CHENG, Zhezhe NIU, Shenglei ZHANG, Yaling BAI, Guolei ZHANG, Jinsheng XU

**Affiliations:** 1 Hebei Clinical Research Center for Chronic Kidney Disease, Hebei Key Laboratory of Vascular Calcification in Kidney Disease, Department of Nephrology, The Fourth Hospital of Hebei Medical University, Shijiazhuang, China; 2 Department of Orthopedics, the Third Hospital of Hebei Medical University, Shijiazhuang, China

**Keywords:** Case-control study, Chronic kidney disease, SFRP1, Coronary artery calcification, Maintenance hemodialysis

## Abstract

This study aimed to examine the association between serum Secreted Frizzled-Related Protein 1 (SFRP1) levels and the presence of coronary artery calcification (CAC) in patients with Stage 5D chronic kidney disease (CKD) undergoing maintenance hemodialysis (MHD). A case-control study was conducted involving 76 patients with CKD Stage 5D undergoing MHD. Participants were stratified into three groups based on Coronary Artery Calcium Scores (CACS): non-calcified (CACS < 100), low-score calcification (100 ≤ CACS ≤ 400), and high-score calcification (CACS > 400). Serum concentrations of SFRP1, hemoglobin, albumin, ferritin, C-reactive protein (CRP), calcium, phosphorus, 25-hydroxyvitamin D, and intact parathyroid hormone were measured. Correlations between biochemical parameters and CAC were evaluated, and multivariate linear regression analysis was employed to identify independent risk factors for CAC. CAC was detected in 60.5 % of the study population. Serum SFRP1 concentrations were significantly elevated in the high-score calcification group compared to both the low-score and non-calcified groups, with the lowest levels observed in the non-calcified group (*p* < 0.01). Positive correlations were observed between CAC and serum levels of SFRP1 levels, phosphorus, age, hemoglobin, alkaline phosphatase, and age. Multivariate linear regression analysis identified serum SFRP1 and phosphorus levels as independent risk factors for CAC in this population (*p* < 0.05). Receiver operating characteristic (ROC) curve analysis demonstrated that serum SFRP1 levels yielded an area under the curve (AUC) of 0.898 (95 % CI: 0.826–0.970, *p* < 0.0001) for predicting CAC, with a Youden index of 0.738 at a cut-off value of 4.242 ng/ml. Elevated serum SFRP1 levels were associated with an increased likelihood of CAC in patients with Stage 5D CKD undergoing MHD. These findings suggest that SFRP1 may represent a potential biomarker for identifying patients at risk for CAC in this population.

## Introduction

Vascular calcification (VC), characterized by abnormal mineral deposition within the vascular wall, represents a common and severe complication among individuals with chronic kidney disease (CKD) [1–4]. Prior research has established a strong association between VC and all-cause mortality in patients with CKD, particularly with increased incidence and mortality related to cardiovascular diseases [5–7]. As a result, strategies aimed at mitigating VC may contribute to improved clinical outcomes in this group.

Secreted frizzled-related protein 1 (SFRP1), a member of the SFRP family, is a secreted glycoprotein containing a cysteine-rich domain. It is highly expressed in fetal renal and cardiac tissues and primarily modulates the Wnt signaling pathway, a mechanism implicated in the pathogenesis of VC [8–13]. SFRP1 has been reported to play a role in the progression of cardiovascular conditions, including coronary atherosclerotic heart disease, myocardial infarction, heart failure, and hypertension [14–18]. Ma et al. reported that inhibition of SFRP1 by WAY-316606 suppressed osteoclastogenesis through dual regulation of the Wnt/β-catenin signaling pathway [19]. Despite these findings, the expression profile of SFRP1 in patients with Stage 5D CKD undergoing maintenance hemodialysis (MHD), as well as its potential role in the pathogenesis of coronary artery calcification (CAC), has not yet been characterized. The present study aimed to investigate the association between serum SFRP1 levels and CAC in this population, with the aim of contributing to improved understanding of potential diagnostic and therapeutic relevance of SFRP1 in the context of VC in individuals receiving MHD.

## Materials and Methods

### Study participants

A total of 76 patients with Stage 5D CKD receiving MHD were enrolled between June and December 2024 from the Hemodialysis Center of the Fourth Hospital of Hebei Medical University.

Inclusion criteria were as follows: (1) age ≥18 years; (2) receipt of regular hemodialysis treatment for a duration exceeding three months, administered three times per week with each session lasting four hours; (3) achievement of target dry weight, as determined by clinical assessment, within the preceding two weeks. Exclusion criteria included (1) age below 18 years or above 80 years; (2) history of malignant tumors, acute infections, acute cardiovascular events, diabetes mellitus, severe malnutrition, or uncontrolled hyperlipidemia; and (3) diagnosis of chronic active hepatitis within the past six months.

The study protocol received ethical approval from the Ethics Committee of the Fourth Hospital of Hebei Medical University (Approval No.: 2024KY001).

### Clinical and serological data

Demographic and clinical characteristics were documented, including age, sex, duration of dialysis, height, weight, urea clearance index (Kt/V), and history of diabetes and cardiovascular disease. Biochemical parameters, including serum albumin (Alb), ferritin, C-reactive protein (CRP), phosphorus, calcium, and alkaline phosphatase, were measured using an automated biochemical analyzer in the clinical laboratory of the Fourth Hospital of Hebei Medical University. Serum SFRP1 levels were quantified using a commercially available enzyme-linked immunosorbent assay (ELISA) kit (Shanghai Biotech Biological Technology Co., Ltd., Shanghai, China).

### Coronary Arterial Calcium Scoring (CACS)

CACS was performed in the Department of Computed Tomography at the Fourth Hospital of Hebei Medical University using a dual-helix computed tomography (CT). The scanning protocol involved image acquisition during the R–R interval of the cardiac cycle, with participants instructed to remain in a calm state and hold their breath. The scanning range extended from the level of the tracheal carina to 1 cm below the diaphragm. CACS was calculated using the Agatston scoring method.

### Grouping

Based on the classification guidelines established by the American Heart Association, participants were categorized into three groups based on their CACS: Group 1: CACS < 100 (non-calcified group), Group 2: 100 ≤ CACS ≤ 400 (low-score calcification group), and Group 3: CACS > 400 (high-score calcification group).

### Statistical methods

Statistical analyses were conducted using SPSS software version 27.0 and GraphPad Prism version 8.0.2. Continuous variables are expressed as mean ± standard deviation (SD). Comparisons between two groups were performed using the independent samples *t*-test, while comparisons among multiple groups were assessed using one-way analysis of variance (ANOVA) or the rank-sum test, as appropriate. Spearman’s rank correlation coefficient was applied to evaluate correlations among multiple variables. Multiple linear regression analysis was used to examine the association between CACS and serum biochemical markers. The diagnostic performance of serum SFRP1 levels in predicting CAC was evaluated using receiver operating characteristic (ROC) curve analysis. Statistical significance was established at *p* < 0.05 for all analyses.

## Results

### General demographic characteristics and clinical biochemical indicators

A total of 76 patients with Stage 5D CKD undergoing MHD were included in the study cohort. Among them, 42 (55.26 %) were male, with a mean age of 53.34 ± 14.69 years and an average duration of dialysis of 8.97 ± 1.12 months. CAC was detected in 46 patients, representing 60.5 % of the study population.

**Fig. 1:**
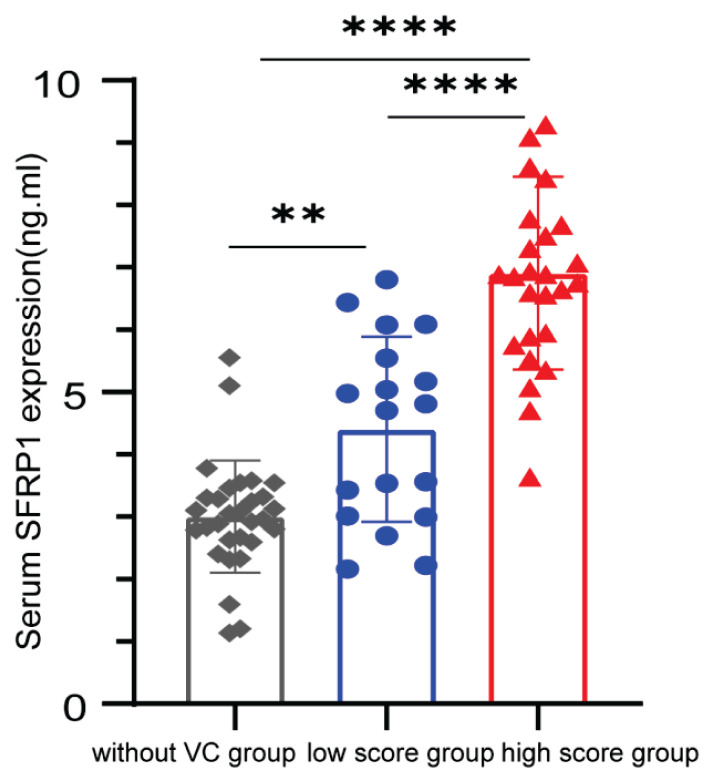
Serum SFRP1 Levels Across Coronary Artery Calcification Groups. Serum SFRP1 concentration were measured by ELISA in 76 patients with Stage 5D CKD Participants were stratified into three groups based on coronary artery calcium scores (CACS): non-calcified (n = 30), low-score calcification (n = 20), and high-score calcification (n = 26). Statistically significant differences in SFRP1 levels were observed between all groups (**p < 0.01, ****p < 0.001).

Statistically significant differences in serum SFRP1 levels were observed among the three patient groups based on CACS. Both the low-score and high-score calcification groups exhibited significantly elevated serum SFRP1 levels compared to the non-calcified group (Fig. 1).

Additionally, significant intergroup differences were observed in CACS, age, sex, history of diabetes, body mass index, urea clearance index, hemoglobin, serum albumin, serum ferritin, CRP, serum phosphorus, parathyroid hormone, 25-hydroxyvitamin D, and alkaline phosphatase levels (Table 1). A progressive increase in CAC severity was associated with rising serum SFRP1 concentrations. Both calcified groups demonstrated significantly higher SFRP1 levels and CACS values compared to the non-calcified group (*p* < 0.05).

Furthermore, serum phosphorus levels were significantly lower in the non-calcified group relative to the calcified groups (*p* < 0.05) (Table 1).

### Correlation between serum SFRP1 levels and clinical indicators

Spearman’s correlation analysis demonstrated a statistically significant positive association between serum SFRP1 levels and age, hemoglobin, serum phosphorus, alkaline phosphatase, and CACS (*p* < 0.05), as presented in Table 2.

### Multivariate linear regression analysis for identifying risk factors influencing CAC in patients undergoing MHD

A multivariate linear regression analysis was conducted using the square root-transformed CACS as the dependent variable. Independent variables included age, body mass index, dialysis duration, hemoglobin, serum albumin, serum ferritin, CRP, serum phosphorus, corrected serum calcium, intact parathyroid hormone, 25﷓hydroxyvitamin D, alkaline phosphatase, and SFRP1 concentration. A stepwise method was employed for variable selection.

The analysis identified serum SFRP1 and phosphorus levels as significant independent factors associated with the CACS. The resulting regression equation was 13.339X1 + 4.259X2 − 32.229, with a coefficient of determination R^2^ of 0.713. The regression model demonstrated statistical significance (F = 15.299, *p* < 0.001).

These findings indicated that elevated serum SFRP1 level was independently associated with the presence and severity of CAC in patients with CKD stage 5D undergoing MHD, suggesting a potential role for SFRP1 as an independent risk factor for vascular calcification in this population (refer to Table 3).

### Predictive Accuracy of Serum SFRP1 for CAC

**Fig. 2:**
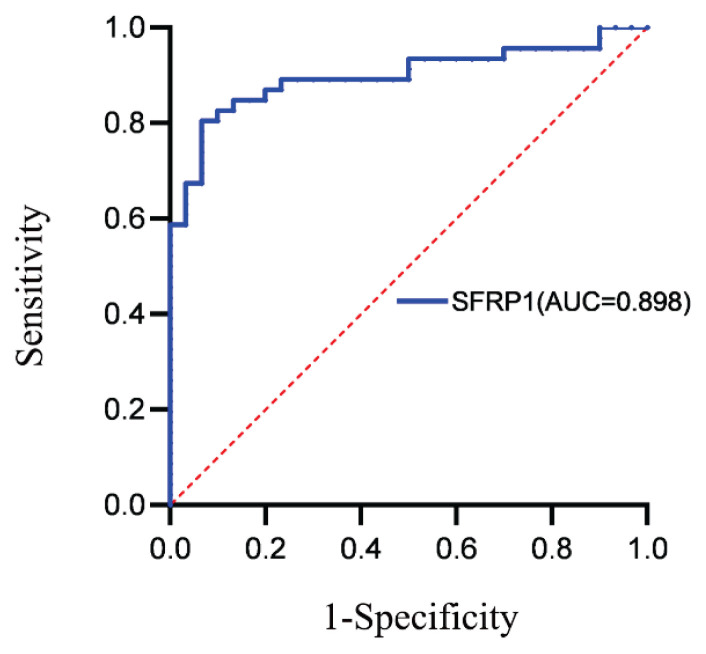
ROC curve of Serum SFRP1 for predicting coronary artery calcification

ROC curve analysis demonstrated that serum SFRP1 had a high diagnostic value in predicting CAC, with an area under the curve (AUC) of 0.898 (95 % CI 0.826–0.970, *p* < 0.0001). The optimal cut-off value for serum SFRP1 levels was determined to be 4.242 ng/ml, exhibiting a sensitivity of 80.4 % and a specificity of 93.3 %, with a Youden index of 0.738 (Fig. 2).

## Discussion

VC is highly prevalent among individuals with CKD and constitutes one of the significant contributors to cardiovascular morbidity and mortality in this population [20,21]. Reported incidence rates of arterial calcification range from 40 % to 60 % in patients with CKD stages 3 to 5, with even higher prevalence observed in those at stage 5D [22].

In the present cross-sectional study, the incidence of CAC among patients receiving MHD was 60.5 %. Elevated serum SFRP1 levels were positively associated with the presence and severity of CAC. A progressive increase CAC severity was observed in conjunction with rising SFRP1 levels. These findings suggested that SFRP1 may function as an independent risk factor for CAC, demonstrating significant predictive value for the development of CAC in patients with stage 5D CKD. Elevated serum SFRP1 concentrations in those undergoing MHD were potentially associated with the presence and severity of CAC.

SFRP1 is a member of the SFRP protein family, which is characterized by the presence of a cysteine-rich domain capable of binding to Wnt ligands [23–25]. To date, five SFRP family members have been identified, designated SFRP1 through SFRP5. Members of this family have been recognized as important modulators in various cardiovascular conditions, including myocardial infarction, cardiac remodeling, and heart failure [26, 27]. Evidence suggests that SFRP1 upregulation may inhibit the proliferation and differentiation of cardiac fibroblasts, thereby reducing the progression of myocardial fibrosis [28]. Additionally, increased SFRP1 expression has been reported in infarcted myocardial tissue following myocardial infarction [29].

Consistent with previous observations, elevated serum levels of SFRP1 were observed in patients with Stage 5D CKD, with significantly higher levels detected in both the low-score and high-score calcification groups compared to the non-calcified group. However, another study reported reduced serum SFRP5 levels in individuals with coronary artery disease (CAD) compared to those without CAD, suggesting a potential protective role of SFRP5 in atherosclerosis through modulation of inflammatory processes [30,31]. These discrepancies in findings may reflect the differential impact of SFRP1 on cardiovascular diseases under varying pathological and physiological conditions, molecular concentrations, and cellular environments [32, 33].

Correlation analysis demonstrated that, among patients with Stage 5D CKD, CACS, age, hemoglobin levels, and serum phosphorus levels exhibited positive associations with serum SFRP1 levels. These findings suggest that increased serum SFRP1 levels were associated with advancing age, elevated hemoglobin levels, and higher serum phosphorus concentrations, all of which corresponded with greater severity of CAC in this population. It is hypothesized that elevated SFRP1 levels may contribute to the activation of the Wnt signaling pathway, thereby promoting the development of CAC in this population.

Multivariate linear regression analysis further indicated that elevated serum phosphorus level represented an independent risk factor for the development of CAC. These observations are consistent with prior research demonstrating a potential link between hyperhosphatemia and the severity of CAC [34]. As such, clinical management strategies aimed at achieving optimal serum phosphorus control may play a critical role in mitigating the progression of CAC in patients with advanced-stage CKD.

ROC curve analysis demonstrated that serum SFRP1 exhibited high diagnostic accuracy for identifying CAC among patients undergoing MHD, indicating its potential utility as a future therapeutic target. However, several limitations should be acknowledged. As a cross-sectional study, causality could not be established, and the relatively small sample size may limit generalizability of the results. Furthermore, the influence of potential unmeasured confounding variables cannot be ruled out. Further research with larger sample sizes and prospective or experimental designs is warranted to validate these findings. Longitudinal investigations tracking dynamic changes in serum SFRP1 concentrations in relation to CAC progression, along with mechanistic studies exploring its role in calcification pathways, remain essential.

## Conclusion

The findings of this study indicated that elevated serum SFRP1 levels may be potentially associated with the presence and severity of CAC in patients undergoing MHD. Serum SFRP1 level was identified as an independent risk factor contributing to the development of CAC. The combined use serum SFRP1 measurement and CACS may facilitate early detection of individuals at increased cardiovascular risk. Such an integrated approach may enable timely clinical intervention, potentially reducing the incidence of adverse cardiovascular outcomes in this population.

## Figures and Tables

**Table 1:** Comparison of clinical and biochemical indicators among patients undergoing maintenance hemodialysis, stratified by coronary artery calcium score

Variable	Non-calcified Group (n = 30)	Low-score calcification group (n = 20)	High-score calcification group (n = 26)	*F/H/X* *^2^*	*P*
*Age (years)*	43.0(36.50~55.25)	58.0(41.25~68.00)	61.0(53.50~69.25)^b^	F=10.05	<0.001
*Male/Female (cases)*	12/18	10/10^a^	20/6	X^2^=7.99	0.018
*History of Cardiovascular Disease, n (%)*	4(13.30)	4(20.00)	8(30.80)	X^2^=2.57	0.277
*History of Diabetes, n (%)*	0(0)	5(25.00)^a^	10(38.46)	X^2^=13.48	0.001
*Body Mass Index (kg/m* *^2^* *)*	21.7(18.6~23.8)	20.8(19.9~23.9)	22.4(20.9~25.5)^b^	H=54.12	<0.001
*Duration on Dialysis (months)*	5.5(3.0~11.6)	4.2(2.7~11.5)	10.8(2.9~14.6)	F=1.41	0.251
*Urea clearance index (Kt/V)*	72.2(67.7~76.4)	74.5(71.1~76.9)^a^	73.2(67.6~74.2)	H=161.0	<0.001
*Hemoglobin (g/L)*	114.0(110.7~120.0)	118.0(114.0~126.0)	120.0(117.0~126.0)^ab^	H=737.53	<0.001
*Serum Albumin (g/L)*	40.56±2.59	40.26±2.88	39.90±2.72^ab^	F=16.65	<0.001
*Serum Ferritin (μg/L)*	665.5(353.1~925.5)	437.8(290.6~795.2)	651.9(406.5~937.4)^ab^	H=1391.8	<0.001
*C-Reactive Protein (mg/L)*	4.1(2.5~7.6)	68.4(3.3~68.4)^a^	4.6(2.6~9.3)	H=46.66	<0.001
*Serum Phosphorus (mmol/L)*	1.77±0.43	1.70±0.42	2.73±0.84^b^	H=44.71	<0.001
*Corrected Calcium (mmol/L)*	2.16±0.17	2.17±0.20	2.18±0.21	F=0.21	0.810
*Parathyroid Hormone (pg/mL)*	218.5(184.9~323.3)	198.4(156.9~230.9)	310.3(213.7~388.7)^ab^	H=2014.73	<0.0001
*25-Hydroxyvitamin D (ng/mL)*	19.9(14.1~25.1)	20.1(14.3~26.0)^a^	23.7(13.1~32.6)	H=7.46	0.024
*Alkaline Phosphatase (U/L)*	96.8(81.1~119.8)	119.5(95.8~150.7)	136.40(111.9~174.2)^b^	H=1386.95	<0.001
*Serum SFRP1 (μg/mL)*	3.01±0.16	4.81±0.42	6.91±0.30^ab^	H=54.03	<0.001
*Coronary Artery Calcium Score (points)*	0	213.0(139.0~241.0)	3456.0(1835.0~4157.0)^ab^	H=8473.23	<0.0001

MHD: Maintenance hemodialysis; SFRP1: Secreted frizzled-related protein 1. Normally distributed measurement data are expressed as mean ± standard deviation, while non-normally distributed data are expressed as median (interquartile range).

a Compared with the non-calcified group, *p* < 0.05 indicates a statistically significant difference;

b Compared with the low-score calcification group, *p* < 0.05 indicates a statistically significant difference.

ab Both the low-score and high-score calcification groups compared with the non-calcified group, *p* < 0.05 indicates a statistically significant difference.

**Table 2:** Correlation analysis between serum SFRP1 levels and clinical indicators

	*r*	*P*
*Age (years)*	0.235	0.041
*Body Mass Index (kg/m* *^2^* *)*	0.091	0.436
*Dialysis Vintage (months)*	0.128	0.271
*Hemoglobin (g/L)*	0.302	0.008
*Serum Albumin (g/L)*	−0.068	0.558
*Serum Ferritin (μg/L)*	−0.215	0.062
*C-reactive Protein (mg/L)*	0.066	0.569
*Serum Phosphorus (mmol/L)*	0.310	0.006
*Corrected Calcium (mmol/L)*	−0.110	0.171
*Parathyroid Hormone (pg/mL)*	−0.144	0.213
*25-Hydroxyvitamin D (ng/mL)*	−0.089	0.444
*Alkaline Phosphatase (U/L)*	0.244	0.034
*Coronary Artery Calcium Score (points)*	0.771	<0.001

SFRP1: Secreted Frizzled-Related Protein 1. *P* < 0.05 indicates a statistically significant difference.

**Table 3:** Multivariate linear regression analysis of factors associated with coronary artery calcium score

Variable	Coefficient of regression	Standard error	Standardized coefficient of regression	*t*-value	*p*-value
*Constant*	−32.229	41.553		−0.776	0.441
*SFRP1 (ng/mL)*	4.259	0.746	0.462	5.71	<0.001
*Age (years)*	0.189	0.104	0.137	1.829	0.072
*Body Mass Index (kg/m* *^2^* *)*	−0.057	0.350	−0.011	−0.164	0.871
*Dialysis Vintage (months)*	0.218	0.192	0.105	1.136	0.26
*Hemoglobin (g/L)*	0.049	0.174	0.022	0.281	0.779
*Serum Albumin (g/L)*	−0.002	0.536	0.000	−0.004	0.997
*Serum Ferritin (μg/L)*	−0.001	0.005	−0.016	−0.230	0.819
*C-reactive Protein (mg/L)*	−0.340	0.215	−0.131	−1.582	0.119
*Serum Phosphorus (mmol/L)*	13.339	2.169	0.465	6.149	<0.001
*Corrected Calcium (mmol/L)*	−11.033	7.468	−0.105	−1.477	0.145
*Parathyroid Hormone (pg/mL)*	0.004	0.013	0.020	0.271	0.788
*25-Hydroxyvitamin D (ng/mL)*	0.210	0.128	0.111	1.636	0.107
*Alkaline Phosphatase (U/L)*	0.050	0.038	0.099	1.317	0.193

Note: Secreted Frizzled-Related Protein 1. *p* < 0.05 indicates a statistically significant difference.

## Abbreviations

SFRP1: Secreted frizzled-related protein 1
CAC: Coronary artery calcification
MHD: Maintenance hemodialysis
